# AAPM Task Group No. 249.B—Essentials and guidelines for clinical medical physics residency training programs

**DOI:** 10.1002/acm2.70111

**Published:** 2025-05-19

**Authors:** Jonathon A. Nye, Joseph P. Dugas, William D. Erwin, David P. Gierga, Darryl G. Kaurin, Stephanie M. Leon, Ho‐Ling Anthony Liu, Zheng Feng Lu, Alphonso W. Magri, Lynn N. Rill, Leah K. Schubert, Beth A. Schueler, Irina Vergalasova, John Vetter, Brian D. Wichman, Amy Shu‐Jung Yu, Dandan Zheng

**Affiliations:** ^1^ Medical University of South Carolina Charleston South Carolina USA; ^2^ Willis‐Knighton Cancer Center Shreveport Louisiana USA; ^3^ UT MD Anderson Cancer Center Houston Texas USA; ^4^ Massachusetts General Hospital Boston Massachusetts USA; ^5^ Northwest Medical Physics Center Lynnwood Washington USA; ^6^ University of Florida Gainesville Florida USA; ^7^ University of Chicago Chicago Illinois USA; ^8^ Upstate Medical Physics Victor New York USA; ^9^ University of Colorado Aurora Colorado USA; ^10^ Mayo Clinic Rochester Minnesota USA; ^11^ Rutgers, The Cancer Institute of New Jersey New Brunswick New Jersey USA; ^12^ University of Wisconsin Madison Wisconsin USA; ^13^ Texas Oncology Round Rock Texas USA; ^14^ Stanford University Stanford California USA; ^15^ University of Rochester Rochester New York USA

**Keywords:** education, medical physics curriculum, residency

## Abstract

The establishment of guidelines and curriculum standards for medical physics residency training is a critical component of setting expectations and competencies for the profession. Since the last publication of these standards, residency training has become integrated into the eligibility criteria for most medical physics certification bodies. The rapid growth of medical physics requires periodic review of the curriculum to remove outdated approaches, update established knowledge, and add emerging technologies. The goal of this document is to provide a reference standard for residency training program directors, training mentors, and residents to guide creation and/or optimization of their program's curriculum. The document is intentionally forward‐looking to capture emerging technological advances including hybrid diagnostic and therapeutic delivery systems and applications of image processing. This document is an update of the current residency training curriculum.

Version history of residency training guidelines

Publication Title

Date

2013 Essentials and Guidelines for Clinical Medical Physics Residency Training Programs—AAPM Report No. 249

2006 Essentials and Guidelines for Hospital‐Based Medical Physics Residency Training Programs—Report No. 90

1992 Essentials and Guidelines for Hospital‐Based Medical Physics Residency Training Programs—Report No. 36

The Work Group on Periodic Review of Medical Physics Residency Training (WGMPRT) would like to acknowledge the authors of past versions of this document as they form the basis for this update. It is the sincere hope of the WGMPRT that this report will continue to be used by the medical physics community to guide training in the profession.

## 1 INTRODUCTION

According to the American Association of Physicists in Medicine (AAPM), medical physics is a branch of physics that applies the concepts and principles of physics to the diagnosis and treatment of human diseases.^1^ Medical physics encompasses four subspecialties: imaging physics, nuclear medicine physics, radiation oncology physics, and medical health physics. This document will concentrate on the essential clinical training requirements for the first three of these subfields.^2^, ^3^, ^4^

Terms such as “shall,” “must,” “should,” and “recommend” are frequently used in this report. The terms “shall” or “must” are used to denote activities the WGMPRT considers as mandatory requirements of the residency program. The terms “should” and “recommend” refer to activities the WGMPRT believes are important but are not mandatory. Recommended activities may be optional because they rely on staff or resources not widely available in all practice settings.

### 1.1 Objectives

The objective of a medical physics residency training program is to educate and train residents to a level of competency sufficient for independent, professional practice on a multidisciplinary team. At the completion of the training program, the resident should be (1) qualified to achieve certification in their specified subspecialties of medical physics; (2) capable of developing and practicing effective strategies to improve and maintain quality care; (3) able to demonstrate a culture of safety, service excellence, professionalism and clinical responsibility; and (4) able to engage in critical thinking and life‐long learning. To accomplish these goals, adequate organization, facilities, staff, patient resources, and educational environments must be provided.

### 1.2 Structure and conduct

Candidates entering residency training shall meet the appropriate didactic training requirements for residency admissions. Clinical training during residency must be conducted over a duration of at least 24 months under the supervision of program staff, of whom a majority hold board certification in the subspecialty areas where training is provided.

#### 1.2.1 Self‐study

All aspects of the training program should be delineated in a self‐study that serves as the primary document governing the conduct of the program and the educational/training pathways from start to completion of the residency. It should demonstrate compliance with all required institutional and extramural standards and policies for the good‐faith function of the program. The self‐study should be continually updated and evolve as the program assesses its practices, strengths, and limitations.

#### 1.2.2 Program structure and models

The training structure and professional experience requirements of the residency training can be acquired through a variety of organizational models. The training model may take on a traditional structure, such as a large academic center, or be composed of a consortium of institutional and/or private members under an affiliation agreement. Adding flexibility to the organizational structure may benefit both the program and trainee; for example, the addition of a training affiliate can provide a wider breadth of staff expertise or expose residents to equipment not available at their home institution. It is recommended that the structure of the training program be disclosed to prospective candidates.

#### 1.2.3 Residency organizational structures

The training program must designate a primary clinical site where the program director is responsible for the residency program. The addition of participating sites may require a training affiliation agreement between the sponsoring institution and participating site with respect to assigned residents, leadership, supervision, and curriculum.^5^ Organization of the training program may take on several models, summarized in Table 1.

**TABLE 1:** Organizational structures for medical physics training (adapted^5^).

Traditional institutional model	Single‐institution academic hospitals, teaching hospitals, and their supporting systems
Limited affiliate model	An association of independent private service groups or public health systems that operate programs independently but hold a single accreditation
Dependent affiliate model	Two or more program sites that share training resources under a single accreditation
Private consultant model	Medical physics service group with training occurring within their healthcare client base

#### 1.2.4 Mixtures of scholarly and clinical training

Residency training programs may choose to include additional time for mentored research, scholarly training, or specialized subspecialty training that is not part of the clinical residency curriculum and may extend the overall training period beyond the minimum 24 months. Some examples may include combining clinical training with a traditional postdoctoral fellowship, as part of graduate training, and/or including specialized training to gain experience on instrumentation or novel techniques. Programs that include nonclinical training should clearly communicate to prospective candidates the structure, expectations, and overall training duration at the time of application.

#### 1.2.5 Salary and benefit expectations

The training program, in collaboration with its participating sites, shall provide all residents with accurate information about financial support and benefits to ensure the successful fulfillment of the expected training and responsibilities of the program. The training program should develop policies and procedures on paid/unpaid leave,^6^ clinical and educational duty hours, and for unexpected events that may interrupt training, such as major medical or natural events. The financial and benefits structure should apply uniformly to all residents who are admitted to the program.

#### 1.2.6 Clinical support expectations

Programs must clearly communicate expectations of any clinical responsibilities, supervision during these activities, and the reporting structure. Policies and procedures should be developed that outline required training, areas of competence, expectations of supervision, administrative approvals from responsible parties, and specific areas of clinical duties prior to a resident taking part in clinical support activities.

#### 1.2.7 Program director

There must be one faculty member designated as the program director who is given the authority and accountability for the program's operations and maintains compliance with accrediting bodies. They must contribute sufficient time to the program to ensure adequate direction. The program director should be provided with

1. salary support for administration that is appropriate for the size of the program; for example, data collected from all programs reported a median FTE of 0.15 (25% quartile = 0.10, 75% quartile = 0.20) and a median FTE/resident of 0.050 (25% quartile = 0.019, 75% quartile = 0.092)—(G. Starkschall, personal communication, April 24, 2023)
2. sufficient time for the administration of the program.

The program director shall be responsible for

1. program administration, operations, and compliance with all applicable requirements;
2. arrangement and oversight of adequate facilities and processes that appointing teaching staff in residency program education at all sites;
3. removal of residents from supervising interactions or learning environments that do not meet the standards of the program;
4. recruitment and appointment of residents that meet the admissions requirements; and
5. periodic evaluations and documentation of resident performance including feedback provided to the residents in a timely manner.

Regarding qualifications, the program director shall

1. be certified in the subfield of medical physics that they are overseeing by an appropriate certifying board and
2. have a minimum of 5 years of full‐time experience practicing in the subfield of medical physics that they are overseeing.

#### 1.2.8 Program steering committee

A program steering committee shall be formed to review the status of the residency training program, track the progress of individual residents, address specific concerns regarding the program training or residents’ performance, and review the program. The committee should consist of the program director, relevant staff, and affiliate partners involved in the training program. It is recommended that a physician and resident representative be involved in select portions of the meeting or select meetings. The steering committee should meet on a schedule adequate to address program matters but not less than two times annually. Residents must have a pathway to communicate their concerns to the committee. All meeting minutes must be recorded and distributed to program staff. Other elements that should be considered under the duties of the steering committee include:

1. Curriculum revision
2. Budget and finances
3. Appointments and departures of program staff
4. Recruitment and admissions
5. Compliance with accreditation authorities
6. Assessment of program strengths and weaknesses.

#### 1.2.9 Program staff and personnel

The program must provide adequate staff and administrative support for the teaching of medical physics in the program's area of specialty. Teaching staff must be qualified in those areas in which they are assigned to instruct and supervise residents, and they must devote the necessary time and effort to the educational program to meet its goals. In addition, staff must maintain a professional and respectful work environment that is conducive to inquiry and scholarship. A commitment to the resident training program on the part of staff is essential to the success of the program.

An adequate staff should include at least two full‐time medical physicists certified by an appropriate certifying board and a radiologist, radiation oncologist, or nuclear medicine physician certified by the American Board of Radiology or its equivalent. Programs can also benefit from access to an administrative assistant or program coordinator. The number of medical physicists who are participating in residency training must be at least one greater than the total number of residents in the program. If available to the program, it would be beneficial to include a radiation biologist on staff.

#### 1.2.10 Institutional support

A strong financial commitment should be made to the residency program that is equitable across the institution and ensures the program can operate at its minimum requirements. These commitments should include support of professional development activities of the staff and residents, access to modern computing and software, and investment in equipment to support the educational objectives. Examples of these items may include costs associated with professional certification, state registration or licensure, teaching and continuing educational resources, professional society membership, attendance of scientific meetings, and support of scholarly activity. In addition, the institution should provide financial support sufficient for regular program activities such as costs associated with maintenance and calibration of equipment, upgrade and maintenance of computer systems, and purchases of simulation and computing software.

The institution sponsoring the medical physics residency program should provide administrative support in terms of funding and space, in addition to clinical and educational resources. Adequate conference room and audiovisual facilities should be provided. Each resident should be allotted office space by the department and be provided with a personal computer with network connections, an email account, telephone, office supplies, and access to a printer, copying machine, and document scanner. The financial support provided should be clearly defined to the resident prior to his or her entry into the residency program (e.g., specified in the resident's appointment letter). An institutional commitment to long‐term funding of the program is essential. Should the program discontinue enrolling residents, there should be a commitment to complete residency training for currently enrolled residents.

#### 1.2.11 Policies and procedures

Programs should establish policies and procedures that govern responsibilities of the resident and program during the training period. The program's policies and procedures should be aligned with their sponsoring institution as well as state, federal, and accreditation requirements. Aspects that should be addressed in these documents include recruitment practices, resident promotion, remediation and dismissal processes, grievance and due process, leave, and expectations of professional behavior. It is the program director's responsibility to counsel, censure, and, after due process, dismiss residents who fail to demonstrate appropriate industry, competence, responsibility, learning abilities, or ethical behavior.

#### 1.2.12 Onboarding process

The program should have a standard process for onboarding residents, for example, (1) designating staff responsible for completing administrative tasks necessary to start in the program; (2) including an orientation schedule introducing the new resident to the program, clinic, staff, and institution; and (3) providing necessary information about the program to start the resident's training efficiently and safely.

New employee information, such as institutional benefits, resources, and policies should be provided. Information specific to the residency program should also be provided to the resident. An example is a residency program handbook, which outlines program requirements, policies, and expectations. The program director should formally meet with the new resident to further communicate program expectations.

The program should provide initial training that is necessary for the resident to safely operate from the first day in the program. This can include training on occupational safety, radiation safety, magnetic resonance safety, infection control, patient privacy, institutional compliance, and the use of relevant clinical software. A dedicated orientation period at the start of the program is useful to provide necessary training and communicate program and institutional expectations. The program should also determine whether new residents need to be licensed, registered, or credentialed based on their state and institutional requirements.

### 1.3 Educational environment

#### 1.3.1 Scholarship, life‐long learning, and conferences

Staff should engage in scholarly activities that support life‐long learning, advancement of clinical services, and innovation in medical physics practice. Resident training should include opportunities for residents to participate in clinical research, provided they support the educational mission and do not distract from the program's curriculum. Examples of these activities may include residents’ participation in commissioning of new technology introduced into the facility, onboarding of new imaging services, and optimization of image quality. Staff and residents are encouraged to present their scholarly activity at local and national conferences.

#### 1.3.2 Documentation of activities

The program should maintain documentation of the resident's attendance at departmental conferences, seminars, journal clubs, or other educational activities. In addition to monitoring residents’ progress, documentation detailing their clinical and didactic activities should be maintained. Residents should maintain these records, but it is the program director's responsibility to periodically review these documents to ensure that they are kept current and that residents are progressing adequately in the program.

#### 1.3.3 Library resources

A sufficient variety of journals, reference books, and other resource materials pertinent to medical physics, basic sciences, diagnostic and interventional radiology, nuclear medicine, and radiation oncology should be provided and be immediately accessible for resident study. Physics residents should also have access to a general medical library and to educational resources available on the Internet. The ability to access these resources must include all hardware to support interacting with these materials, such as audio and video attachments.

#### 1.3.4 Access and use of medical information

Residents should be given sufficient access to medical data as needed to perform training and clinical tasks described in the program's self‐study. Residents must complete all necessary compliance training prior to access, and access should be limited to a duration needed to reasonably complete the task.

#### 1.3.5 Remote learning and teaching

##### Definition and scope

Remote learning and teaching refer to an educational activity which occurs when the trainee and the instructor are not physically in the same location. It can be synchronous, such as via a conference call, or asynchronous, such as via a prerecorded video or a project assignment. Remote learning does not occur face‐to‐face; therefore, certain types of technology are involved. Remote opportunities should be of a quality comparable with in‐person or onsite experiences for which they are substituting. Learning outcomes of the remote opportunities should be equivalent to that of in‐person or onsite experiences and should be closely monitored and evaluated.

##### Feasibility of remote learning in medical physics residency training

Medical physics residency training cannot be completed solely based on remote learning. Didactic courses, teaching of soft skills, clinical conferences, and seminars are good options to utilize remote learning. Hands‐on training and assessment should be conducted in person. Clinical observations are difficult to achieve remotely. Considering resident supervision, training through clinical tasks performed under general supervision may be conducted remotely or using the combination of remote and in‐person instruction.

##### Technical considerations

If remote learning is utilized, necessary hardware and software should be provided to residents to enable a high‐quality experience. They include computing equipment that provides a stable and secure network, software and remote access needed to perform assigned duties, and video‐conferencing equipment and software.

#### 1.3.6 Didactic knowledge requirements

Upon entering a medical physics residency program, the candidate will have didactic knowledge of medical physics comparable with that obtained from a CAMPEP‐accredited medical physics graduate program. This is accomplished most directly by accepting graduates from accredited medical physics graduate or certificate programs into the residency program.

Alternatively, graduates of nonaccredited medical physics graduate programs and graduates of physics or related graduate programs may be accepted into a medical physics residency program if they can demonstrate they have successfully completed the undergraduate and graduate didactic prerequisites. The content of these courses should comply with the recommendations provided in AAPM Report 365.^7^

### 1.4 Professional integrity

#### 1.4.1 Ethics curriculum for medical physics residency programs

A residency training program must include instruction on professional ethics with specific attention given to ethical decision making in medical physics clinical practice. Guidance for ethical training of medical physicists has been provided in AAPM Report No. 159,^8^ which includes historical ethical perspectives, principles, encounters and dilemmas, professional conduct, clinical practice ethics, research ethics, and education ethics. Additional resources such as the most current version of the AAPM Code of Ethics Professional Policy,^9^ teaching resources from the AAPM, and other relevant professional societies (such as the ABR/ACR/RSNA/AAPM/ASTRO/ARR/ARS Online Modules on Ethics and Professionalism^10^), resources from fields outside of medical physics, as well as institutional resources are also available. Other forms of effective teaching methods such as case study discussions or self‐reflection should be considered.

#### 1.4.2 Professionalism and leadership

As a member of a diverse and multidisciplinary environment, medical physicists must engage in professional conduct that focuses on elements of behavior and accountability, cultural proficiency, teamwork, and professional growth. Integrated with the resident's technical skill development, it is essential that the program support development of professional skills and behaviors to achieve the common goal of delivering the highest quality patient care. The AAPM provides within its Code of Ethics general guidelines for professional conduct as it applies to medical physicists.^9^ Training should be provided that adheres to the expectation of the profession and the individual program's institutional professional policies.

Leadership is a critical element to professional competency and should be incorporated into the program curriculum. It is recommended that programs consult resources developed by the AAPM's Medical Physics Leadership Academy to assist with developing opportunities in their training curriculum.^11^

An additional professional skill that merits special attention is communication. Residents should be given opportunities to practice, refine and assess their communication skills for interactions with physicists, physicians, clinical staff, administrators, patients, and the public. The medical physicist is a critical component of the healthcare team, providing expertise on the applications of ionizing radiation in medicine, on patient safety, and on radiation risk, and should be well prepared to communicate this expertise. The role of the medical physicist in the care team is currently evolving and often requires direct medical physicist‐patient interactions. Residents should be prepared for these interactions through dedicated patient‐communication training.

#### 1.4.3 Diversity, equity, and inclusion

It is essential that medical physics residency programs be committed to fostering collaborative environments of equity, diversity, and inclusion.^12^ Programs should offer or provide access to formal training promoting workplace wellness and cultural competence to its program staff and residents. Examples may include training in balanced and unbiased practices during resident recruitment and admissions to ensure an equitable process for all applicants. Clinical training may include conversations and strategies to ensure equitable patient care such as providing education on healthcare disparities and social determinants of health. It is the program's responsibility to safeguard an environment free of any kind of harassment or discrimination.

#### 1.4.4 Role of medical physicists in providing quality care

When training future generations of medical physicists, it is important to incorporate a visionary perspective of the evolution of our field. This is the goal of the Medical Physics 3.0 initiative, which strives to shape and more explicitly define the role of medical physicists in providing quality care by “pushing into new territories of scholarship and practice in medicine.^13^” Medical Physics 3.0 has generated a list of “smart” aspirations for medical physicists to emulate in their careers moving forward. Residency training programs should evole their training curriculum as the medical physics field and the role of medical physicists evolve.

### 1.5 Medical physics educational competencies

Medical physics residency training programs must develop a written curriculum and areas of competence for their residents that clearly delineate expectations of resident performance, behavior, and training schedule. These expectations must be provided at the time the resident enters the program and periodically reviewed through a formalized evaluation process. Following points of evaluation, residents should be given timely written feedback regarding their performance. If their performance is deemed inadequate, a course of action for remediation should be developed in a timely manner.

Specific recommendations for curricula in diagnostic imaging, nuclear medicine, and radiation oncology physics are detailed in Sections 2.1, 3.2, 4.2. For all listed items, residents shall understand relevant (1) theory of operation and basic physics, (2) safety precautions and regulations, (3) practical usage procedures and considerations, and (4) interpretation of results. Development and evaluation of competency goals should be applied consistently over the course of the program and span the six core competencies specified by the American Board of Medical Specialties and the Accreditation Council for Graduate Medical Education.^14^ The six core competencies in medical physics resident education are:

1. Patient care and procedural skills
2. Medical knowledge
3. Interpersonal and communication skills
4. Professionalism
5. Practice‐based learning and improvement
6. Systems‐based practice.

Programs can incorporate these core competencies as part of the evaluation process. See Appendices A and B for examples.

#### 1.5.1 Progression of supervision

The program staff should provide a systematic course of instruction, assessment, and evaluation that encourages progressive, supervised resident responsibility for patient care. These responsibilities must include performance of commonly accepted clinical physics procedures in all aspects of the respective subspecialty scope of practice. A log of these events should be reviewed periodically by the program director and program steering committee and be available for external review of the program. Progression of supervision should align with the categories described in the AAPM medical physics practice guidelines^15^ starting with personal supervision and progression to direct supervision with a goal to achieve general supervision when appropriate.

### 1.6 Assessment and evaluation

#### 1.6.1 Resident evaluation

Programs must form a standardized process that documents the assessment and evaluation of residents’ educational performance and progress. As part of this process, the resident must meet with the program director periodically. Documentation of the meeting minutes shall be accessible to the resident. Monthly meetings of the resident with the program director are recommended. In addition, the resident should meet periodically with the training supervisor (e.g., biweekly).

The resident must be evaluated upon completion of an area of study or clinical rotation. Over the course of this period, resident performance and progress should be assessed periodically by the program faculty. There are currently no standardized criteria or scales for assessing resident performance, but examples of common assessments tools are listed below.

1. Workplace‐based performance assessments: Direct observation of the resident in a workplace or clinical environment or an assessment of performance provided by one or more program staff who have interacted with the resident during a specific period.
2. Written tests: A list of items, such as multiple‐choice questions, one‐best answer, or other item types that surveys an area of clinical knowledge and skills.
3. Oral examination: A face‐to‐face meeting between a resident and one or more program staff where focused questions are presented to assess critical thinking, problem solving, judgment, and ethics.
4. Objective clinical performance assessments: The resident is presented with a real or simulated workplace task and asked to complete the task under observation.
5. Narrative instruments: A self‐directed exercise that may include generation of a report demonstrating specific subject knowledge.
6. Clinical activity log: A summary of tasks performed by the resident such as annual measurement of instrumentation output.

Evaluations are completed with information gathered from one or more of the example assessment methods described above. These are completed either by the training mentor, program director, or as part of a clinical competence committee. Evaluation tools may have several evaluation methods integrated into a single document:

1. Rater or competency checklist: Bulleted items focused on areas of competency and medical physics knowledge.
2. Entrustable professional activity^16^: Ability to competently and safely perform a clinical task and be considered proficient such that the activity can be entrusted to the resident.
3. Milestone^17^: A continuous evaluation structure that tracks resident progress from novice to expert and is revisited periodically to evaluate progress along the continuum.
4. 360 evaluation^18^: Feedback mechanism available to supporting staff in different service lines that participate in clinical operations including administrators, educators, and peers.

The program director should document any prior training from another institution that is being used to satisfy the training requirements of the program. A resident's prior training may be a reason to modify the training content of a rotation, for example, by providing the resident with more advanced training.

#### 1.6.2 Staff and program evaluation

The training program should provide a means by which residents can give feedback regarding the performance of their clinical supervisors, training mentors, and quality of training in a rotation. Measures should be taken to ensure the confidentiality of any individual resident's comments such as aggregating reports. Staff evaluations should be reviewed by the program director as they are submitted. If an area of concern is identified, a plan of remediation should be developed in consultation with the steering committee.

Periodically and at the completion of the program, the resident should be given an opportunity to evaluate the training program in its entirety. Evaluation of program staff should include a discussion of their teaching abilities, commitment to the training program, clinical knowledge, and professionalism. These program evaluations should be reviewed by the program steering committee; items of concern should be identified and addressed.

#### 1.6.3 Internal program review

As part of the program oversight by the program director and/or steering committee, a comprehensive annual review should be conducted that identifies strengths, weaknesses, opportunities, and threats to the program. The review may be conducted by an internal committee. In addition, the program should review their self‐study with current standards from their respective accreditation body and make updates as needed. It is recommended that feedback be collected from residents who have completed the program.

## 2 GUIDELINES FOR IMAGING PHYSICS RESIDENCY TRAINING PROGRAMS

### 2.1 Training content

Training in the clinical and technical areas of imaging physics should include the following: physical principles and procedures involved in the production of clinical images, methods of image evaluation, techniques for optimizing radiation exposure, methods for calculating organ doses and estimating risk, calibration and monitoring of imaging equipment, and radiation safety procedures. These areas of medical physics knowledge should include education and experiences with emerging technologies and applications of techniques that may form the basis for future imaging procedures. Areas of competence should reference accepted clinical practices and techniques which may include but are not limited to published reports by international and AAPM committees or work groups, peer‐reviewed literature, textbooks, and consensus documents from leading professional organizations.

### 2.2 Facilities

Adequate space and appropriate resources must be available for the conduct of a good medical physics practice and clinical training program. At a minimum, the following resources are required to ensure that adequate imaging modalities are available for observation and training:

1. Digital radiography systems
2. Digital fluoroscopy systems with optional access to image intensifier fluoroscopy systems and
  - General fluoroscopy under‐ and over‐table configurations
  - C‐arm systems including both single‐plane and biplane versions
  - Mobile C‐arm system
3. Full‐field digital mammography systems and,
  - Tomosynthesis system
  - Stereotactic biopsy system
  - Access to computer aided detection software.
4. Computed tomography (CT) scanner
5. Magnetic resonance imaging (MRI) systems at two different field strengths
6. Ultrasound (US) imaging system
7. Single‐photon emission CT (SPECT) and positron emission tomography (PET) system.
8. An electronic imaging management and reporting system such as a picture archiving and communications system (PACS) and radiology information system (RIS).

If any of the above modalities are not available at the program site, the program must provide the clinical training at another site which has an affiliation agreement with the program site. Research imaging equipment may be substituted for systems in clinical operation only when substitutes are substantially equivalent to commercial clinical equipment for imaging humans.

### 2.3 Clinical resources

The training program in imaging physics must provide a sufficient volume and variety of patients for a comprehensive resident experience. The number of diagnostic or interventional examinations per year should be sufficient for a resident to become competent in imaging physics. It would be satisfactory to gain these experiences through arrangements made with other institutions for the resident to receive training on specialized equipment or diagnostic procedures that are not performed at the host training facility.

### 2.4 Training requirements

Upon completing an imaging physics residency program, the resident should demonstrate proficiency and competency in the following areas:

1. Assessments of imaging systems
2. Understanding and technical knowledge of imaging systems
3. Safety culture
4. Professional interaction and leadership
5. Components of clinical practice
6. Professional consultation
7. Clinical procedure observations
8. Risk management
9. Regulatory compliance
10. Practice accreditation.

The program should develop a written list of objectives for or expectations of residents that should be presented to and discussed with residents. A list of specific areas of competence recommended for clinical medical physics in diagnostic imaging is provided below. Sample templates for presenting these objectives are provided in Appendix B; however, at this time, there are no standardized criteria or scales for evaluating residents’ performance during their clinical rotations. Participation in clinical research in imaging physics is recommended.

Under Section 2.5, areas of competence marked as electives are topics that may not be available to all training programs. Clinical areas of competence developed in these topic areas are considered optional. However, there may be a need to develop areas of competence based on medical knowledge required to meet a level of understanding sufficient for the resident to complete their chosen subspecialty certification.

### 2.5 Expected areas of competence for clinical medical physicists in imaging

#### 2.5.1 General radiography

##### Understanding the imaging chain

1. x‐Ray tube and filtration
  - Spectrum characteristics
  - Focal spot sizes
  - Heat loading
  - Filtration characteristics
  - Collimation
2. Scatter rejection
  - Grids
  - Air gap
  - Slot scanning
3. Technique selection and exposure control
  - Fixed techniques and charts
  - Automatic exposure control
4. Detector technology
  - Direct technology
  - Indirect technology
  - Charged‐coupled device
  - Computed radiography

##### Performance assessment

1. Output
2. Collimation and x‐ray beam alignment
3. Automatic exposure control
4. kVp accuracy and waveform
5. Timer accuracy
6. Half‐value‐layer
7. Exposure lrinearity
8. Exposure Reproducibility
9. Exposure indicator accuracy
10. Technologist quality control program

##### Image quality

1. Image quality metrics
2. Image preprocessing and corrections
3. Image postprocessing
4. Exposure indices
5. Image artifacts

##### Safety

1. Room shielding design
2. Entrance skin dose
3. Patient shielding
4. Regulations and policies for patient holders
5. Room surveys and inspections
6. Diagnostic reference levels and achievable doses

##### Clinical

1. Optimization
2. Exam performance
3. Fixed and portable geometry considerations
4. Repeat/reject analysis
5. Considerations for pregnant and pediatric patients

##### Electives

1. Digital radiography tomography
2. Dental units including intraoral and panoramic
3. Dedicated spine imaging
4. Dual energy projection radiography
5. Dual energy x‐ray absorptiometry

#### 2.5.2 Angiography and fluoroscopy

##### Understanding the imaging chain

1. X‐Ray tube design and spectrum characteristics
2. Dose monitoring system
3. Image intensifier
4. Flat panel detector
5. Magnification modes
6. Grids
7. Automatic exposure rate control
8. Spectral filtration
9. Wedge filters
10. System configuration and geometry
11. Image display devices

##### Performance assessment

1. Collimation and x‐ray beam alignment
2. Automatic exposure rate control assessment
3. High‐contrast resolution
4. Low‐contrast resolution
5. kVp accuracy and waveform
6. Half‐value‐layer
7. Reproducibility
8. Output and accuracy
9. Displayed air kerma accuracy

##### Image quality

1. Image quality metrics
2. Fluoroscopy and recorded imaging
3. Temporal resolution
4. Artifacts
5. Dose and image quality optimization
6. Image processing

##### Safety

1. Room shielding design
2. Shielding and scatter surveys
3. Entrance skin dose
4. Peak skin dose
5. Occupational fluoroscopy safety program
6. Regulatory requirements and policies
7. Patient safety management program

##### Clinical

1. Protocol optimization and operating parameters
2. Procedure observations and equipment utilization
3. Procedure entrance exposure rates
4. Contrast agents and injectors
5. Communication of procedure risks and follow‐up procedures
6. Considerations for pregnant and pediatric patients

##### Electives

1. Cone beam fluoroscopy or CT (O‐Arm)
2. Advanced fluoroscopy guided interventional systems including robotics
3. Lithotripter
4. Mini C‐Arms

#### 2.5.3 Computed tomography

##### Understanding the imaging chain

1. Collimation
2. x‐Ray filtration
3. Automatic exposure control
4. (Multi‐) Detector configuration
5. Axial, helical, and cone beam acquisition geometry
6. Image reconstruction

##### Performance assessment

1. Table travel accuracy
2. Slice position accuracy
3. Slice sensitivity profile
4. Radiation beam width
5. Displayed CT dose index accuracy
6. CT number accuracy
7. Acquisition monitor
8. Contrast‐to‐noise ratio
9. Signal‐to‐noise ratio

##### Image quality

1. High‐contrast resolution
2. Low‐contrast resolution
3. Noise
4. Image processing
5. Uniformity
6. Artifacts

##### Safety

1. Patient dose monitoring and management
2. Size specific dose estimate and water equivalent diameter
3. Dose notification and alert values

##### Clinical

1. Protocol optimization and protocol management
2. Contrast agents and injectors
3. Communication of procedure risk
4. Procedure doses
5. Considerations for pregnant and pediatric patients

##### Electives

1. Dual‐energy systems
2. Flat panel systems
3. Specialized cone beam systems
4. Photon counting systems

#### 2.5.4 Ultrasound

##### Understanding the imaging chain

1. Transducer
2. Beam formation
3. Interactions of US waves with matter
4. A‐Mode, B‐Mode, and M‐Mode
5. Doppler US
6. Harmonic imaging
7. Compound imaging
8. Shear wave elastography

##### Performance assessment

1. System sensitivity
2. Geometric accuracy
3. Transducer element check
4. Display performance assessment
5. Doppler performance assessment
6. Spatial Resolution: Axial, lateral, elevational

##### Image quality

1. Image uniformity
2. Contrast resolution
3. Lesion detectability
4. Speckle noise
5. Artifacts survey

##### Safety

1. Bioeffects
2. Thermal and mechanical index
3. Acoustic output metrics (spatial average/temporal average intensity, spatial peak/temporal average intensity, spatial peak/pulse average intensity, spatial peak/temporal peak intensity)
4. Safe clinical uses of diagnostic US

##### Clinical

1. Interpretation of artifacts
2. US guided procedures and biopsy
3. Considerations for pregnant and pediatric patients

##### Electives

1. Three‐dimensional (3D) US imaging
2. Automated whole breast US
3. Contrast enhanced US
4. High intensity focused US

#### 2.5.5 Mammography

##### Understanding the imaging chain

1. Collimation
2. Spectral filtration
3. Target‐filter combinations
4. Focal spot sizes
5. Automated exposure control
6. Detector configuration
7. Grids
8. Magnification
9. Compression

##### Geometric configurations

1. Full‐field digital mammography
2. Tomosynthesis and reconstruction
3. Stereotactic or tomo‐breast biopsy systems

##### Performance assessment

1. Image quality evaluation
2. System evaluation
3. Collimation
4. kVp accuracy
5. Half value layer
6. Automatic exposure control
7. Output
8. Entrance skin exposure and average glandular dose
9. Reading room and workstation display
10. Stereotactic localization accuracy

##### Image quality characteristics

1. Image quality metrics
2. Image processing
3. Artifacts and uniformity

##### Safety

1. Device hazards
2. Entrance Skin Dose
3. Average Glandular Dose

##### Clinical

1. Protocol optimization and operating parameters
2. Procedure observations and equipment utilization
3. Considerations for pregnant patients

##### Electives

1. Contrast Enhancement
2. Alternative Imaging Geometries

#### 2.5.6 Magnetic resonance imaging

##### Understanding the imaging chain

1. Static Magnetic Field
2. Gradient coil specifications including strength, slew rate and linearity
3. Radiofrequency (RF) coils
4. Signal and contrast generation
5. Spatial encoding
6. Pulse sequences
7. Pulse sequence options including inversion recovery, fat suppression, spatial saturation, flow compensation
8. Acceleration methods
9. k‐space and Image reconstruction
10. Intensity uniformity correction
11. Geometric distortion correction

##### Performance assessment

1. Siting‐related tests
2. Magnetic fringe field mapping
3. System inventory
4. General system checks
5. Magnetic field homogeneity
6. Magnetic field drift
7. Geometric accuracy
8. Table position accuracy
9. Transmitter gain and attenuation
10. Image intensity uniformity
11. Slice‐position accuracy
12. Slice‐thickness accuracy
13. RF coil testing
14. Technologist quality control program

##### Image quality

1. Spatial resolution
2. Contrast
3. Signal‐to‐noise ratio
4. Artifacts
5. Scan time

##### Safety

1. Specific absorption rate (SAR)
2. Facility design and requirements
3. MRI safety training and policies
4. Static magnetic field hazards
5. RF burns, peripheral nerve stimulation, and acoustic noise
6. Implanted devices, operating modes, SAR, and B1+rms limits
7. MR safe, MR conditional, and MR unsafe
8. Quench

##### Clinical

1. Protocol optimization and protocol management
2. Contrast agents and injectors
3. Communication of procedure risks
4. Considerations for pregnant and pediatric patients

##### Electives

1. Open field systems and permanent magnets
2. Breast MRI
3. Ultrahigh‐field magnet systems
4. Advanced MRI methods including functional MRI, MRS, perfusion, and diffusion
5. Interventional and interoperative MRI

#### 2.5.7 Nuclear medicine and positron emission tomography

##### Understanding the imaging chain

1. Scanner components and configurations
2. Collimation and event processing
3. Projections and sampling
4. Acquisition modes
5. Reconstruction algorithms

##### Performance assessment

1. Image quality evaluation
2. System sensitivity
3. System data corrections

##### Image quality characteristics

1. Image quality metrics
2. Quality phantoms
3. Image artifacts

##### Safety

1. Quality control program and oversight
2. Regulation and medical use of radioactive materials
3. Nonimaging devices
4. Internal dosimetry
5. Hot lab operations

##### Clinical

1. Common diagnostic protocols and indications
2. Common therapeutic protocols and indications
3. Considerations for pregnant and nursing patients
4. Considerations for pediatric patients

#### 2.5.8 Data science and informatics

##### Receiver operator characteristic performance

##### Image registration and similarity metrics

##### Image processing

1. Fusion display
2. Maximum/Minimum/Average intensity projection
3. Contrast enhancement

##### Quantitative imaging and signal mapping

##### Imaging statistics

1. Signal‐to‐noise ratio
2. Contrast‐to‐noise ratio
3. Statistical distributions

##### Computer‐aided detection and diagnosis

##### Display performance and viewing conditions

1. Measures of luminance and illuminance
2. Just Noticeable Difference Index and Barten model
3. DICOM grayscale standard display function
4. Acquisition and workstation display periodic quality control and annual assessments

##### Enterprise storage and display systems

1. Picture archiving and communication systems
2. Network attached storage servers, distributed licensing, and client display

##### DICOM communications and format standards

##### Electronic medical systems

1. Electronic medical records
2. Radiology Information System
3. Communication standards

##### Applications of artificial intelligence and machine learning

#### 2.5.9 General safety and radiation protection

##### Regulatory agencies, jurisdiction, and publications

##### Advisory bodies

##### Protection standards and practices

1. Dose quantities and units
2. Regulatory limits and usage regulations
3. Radiation safety program
4. Historical accidents
5. Guidelines and safety principles
6. Safety program for non‐radiology staff and physicians

##### Shielding

  1. Design goals
  2. Facility design
  3. Barrier thickness calculations
  4. Radiation survey and evaluation
  5. Considerations for hybrid systems

##### Patient protection practices

1. Regulation limits and badging
2. Considerations for pregnant women
3. Fetal and organ dosimetry
4. Dose monitoring systems
5. Radiation dose and image quality optimization
6. Consideration for the pediatric population

##### Diagnostic reference levels and achievable dose

##### Radiation risks

1. Stochastic
2. Deterministic
3. Models of risk assessment
4. Fetal considerations

##### Radiation detection and monitoring devices

1. Gas ionization chambers
2. Solid state systems
3. Scintillation systems
4. Personal dosimetry systems

## 3 GUIDELINES FOR NUCLEAR MEDICINE PHYSICS RESIDENCY TRAINING PROGRAMS

### 3.1 Training content

Training programs should provide instructional opportunities on the calibration and monitoring of nuclear medicine instrumentation, the safe handling and distribution of radioactivity, regulatory procedures and the processes governing a radiation safety program, opportunities to observe clinical imaging workflow and reporting, and computing resources for image processing and analysis. Areas of competence should reference accepted clinical practices and techniques which may include but are not limited to published reports by international and AAPM committees or work groups, peer‐reviewed literature, textbooks, and consensus documents from leading professional organizations.

The growing number of commonalities between imaging and nuclear medicine physics practice has created an opportunity for residency programs to offer combined imaging and nuclear medicine physics residencies in a highly efficient manner. Utilizing the commonalities between these subspecialties may allow a program to provide a nuclear medicine physics residency over a minimum of a 1‐year period following completion of an imaging residency program. Details on such an approach are provided in Appendix C.

### 3.2 Facilities

Adequate space and appropriate resources must be available for the conduct of a good clinical physics practice and training program. Systems and equipment that must be accessible to a nuclear medicine residency program include

1. Imaging Systems
  - A minimum of two gamma cameras
  - SPECT system
  - SPECT/CT system
  - PET/CT system
  - A computer for image analysis
2. Nonimaging instrumentation
  - Dose calibrator
  - Thyroid probe
  - Well counter
3. Radiopharmacy or hot lab

Research imaging equipment may be substituted for systems in clinical operation only when substitutes are substantially equivalent to commercial clinical equipment for imaging humans.

### 3.3 Clinical resources

The training program in nuclear medicine physics must provide a volume and variety of clinical procedures sufficient for a comprehensive resident experience. The number of nuclear medicine procedures and treatments per year should be sufficient to permit a resident to become competent and proficient in nuclear medicine physics. In some practice settings, access to certain diagnostic or therapeutic protocols may be limited or unavailable, in which case programs are encouraged to seek out collaborative partners who can supplement training in these areas.

### 3.4 Training requirements

Upon completing a nuclear medicine physics residency program, the resident should demonstrate proficiency and competency in the following areas:

1. Assessments of nuclear medicine imaging and nonimaging systems
2. Understanding and technical knowledge of nuclear medicine imaging and nonimaging systems
3. Safety culture
4. Professional interaction and leadership
5. Components of clinical practice
6. Professional consultation
7. Clinical procedure observations
8. Risk management
9. Regulatory compliance
10. Practice accreditation

The program should develop a written list of objectives for or expectations of residents that should be presented to and discussed with residents. A list of specific areas of competence recommended for clinical medical physics in nuclear medicine is provided below. Sample templates for presenting these objectives are provided in Appendix B; however, at this time, there are no standardized criteria or scales for evaluating residents’ performance during their clinical rotations. Clinical research in nuclear medicine physics is recommended.

Under Section 3.5, areas of competence marked as electives are topics that may not be available to all training programs. Clinical areas of competence developed in these topic areas are considered optional. However, there may be a need to develop areas of competence based on medical knowledge required to meet a level of understanding sufficient for the resident to complete their chosen subspecialty certification.

### 3.5 Expected areas of competence for clinical medical physicists in nuclear medicine

#### 3.5.1 Imaging systems

##### Planar imaging

1. Understanding the imaging chain
  - Gamma camera components
  - Anger logic
  - Energy window selection
  - Collimation
  - Matrix size and zoom
  - Acquisition modes
  - Data storage
2. Performance specifications and assessments
  - Energy resolution
  - Radionuclide peaking and energy linearity
  - Spatial resolution
  - Uniformity
  - System sensitivity
  - Count rate performance
  - Multiple‐window spatial registration
  - Opposed‐head registration
  - Crystal hydration
3. Corrections
  - Energy
  - Spatial linearity
  - Uniformity
4. Quantification
  - Geometric mean
  - Time‐activity curve analysis
  - Regions of interest calculations
5. Planar Quality Control
  - Peaking
  - Uniformity
  - Spatial resolution
  - Correction maps

##### SPECT and SPECT/CT

1. Understanding the imaging chain
  - Camera configurations
  - Orbits
  - Acquisition of projections
  - Reconstruction algorithms and filtering
2. Performance specifications and assessments
  - Uniformity
  - Spatial resolution
  - Contrast resolution
  - Hybrid image coregistration
3. Corrections
  - Multihead registration
  - Center of rotation
  - Attenuation
  - Scatter
  - Resolution or collimation model
  - SPECT and CT gantry alignment
4. SPECT quality control
  - Center of rotation

##### PET/CT

1. Understanding the imaging chain
  - Positron physics
  - Coincidence detection
  - Time‐of‐flight
  - Event types
  - Data storage types
  - Scintillator material and detector configuration
  - Image reconstruction
2. Performance specifications and assessments
  - Spatial resolution
  - Sensitivity
  - Count rate performance
  - Scatter fraction
  - Image quality and contrast recovery
  - Accuracy of corrections
  - Hybrid image coregistration
  - Standardized uptake value accuracy
  - Image uniformity
3. Corrections
  - Normalization
  - Scatter estimation and modeling
  - Random coincidences
  - Attenuation estimation
  - Dose calibrator cross calibration
  - Count rate performance and dead time
  - Decay correction
  - Radionuclide specific corrections
  - Hybrid PET and CT gantry alignment
4. Quantification
  - Partial volume effects
  - Spatial and temporal sampling
  - Standardized uptake value
5. PET Quality Control
  - Normalization and gain calibration
  - Energy peaking
  - Coincidence timing
  - Inspection of sinogram
  - Uniformity
  - Standardized Uptake Value accuracy
  - Well counter cross‐calibration
  - Quality control phantoms and radioactive sources
  - Interpretation of quality control reports

##### CT Scanner (with SPECT or PET)

1. Understanding the imaging chain
  - Collimation
  - x‐Ray filtration
  - Automatic exposure control
  - (Multi‐) Detector configuration
  - Axial, helical, and cone beam acquisition geometry
  - Image reconstruction
2. Performance specifications and assessments
  - Table travel accuracy
  - Slice position accuracy
  - Slice sensitivity profile
  - Radiation beam width
  - Displayed CT dose index accuracy
  - CT number accuracy
  - Acquisition monitor
  - Contrast‐to‐noise ratio
  - Signal‐to‐noise ratio
3. Image Quality
  - High‐contrast resolution
  - Low‐contrast resolution
  - Noise
  - Uniformity
  - Attenuation map
  - Intermodality coregistration
  - Gantry alignment
4. Safety
  - Patient dose monitoring and management
  - Size specific dose estimate and water equivalent diameter
  - Scatter surveys
  - Dose notification and alert values
5. Clinical
  - Protocol optimization and protocol management
  - Contrast agents and injectors
  - Communication of procedure risk
  - Procedure doses

##### Electives

1. PET/MR
2. Dedicated cardiac SPECT(/CT)
3. Dedicated breast planar systems
4. Dedicated breast PET systems
5. Total‐body volumetric SPECT systems
6. Large axial field‐of‐view PET systems
7. Pixelated planar gamma camera and SPECT systems
8. Quantitative SPECT

#### 3.5.2 Nonimaging devices

##### General performance characterization

1. Energy resolution
2. Linearity
3. Constancy
4. Accuracy
5. Geometric efficiency
6. Temporal resolution and dead time
7. Counting efficiency
8. Counting statistics and error propagation
9. Chi‐squared
10. Minimum detectable activity
11. Correct selection and use of instrumentation
12. Energy calibration
13. Quality control program

##### Survey meters

1. GM counters
2. Ion chambers
3. Scintillators

##### Uptake probes and well counters

##### Dose calibrators

##### Personal dosimeters

##### Electives

1. Solid state detectors and probes
2. Liquid scintillation counter

#### 3.5.3 General safety and radiation protection

##### Regulatory agencies, jurisdiction, and publications

##### Advisory bodies

##### Radioactive material transport, receiving, and disposal

##### Protection standards and practices

1. Dose quantities and units
2. Regulatory limits and usage regulations
3. Radiation safety program
4. Quality management
5. Historical accidents
6. Guidelines and safety principles

##### Medical use regulations

1. Authorized user
2. Written directive
3. Medical event

##### Shielding

1. Exposure rate constants
2. Shielding design goals
3. Facility design
4. Radiation survey and evaluation
5. Consideration for hybrid systems

##### Diagnostic reference levels and achievable dose

##### Radiation risks

1. Stochastic
2. Deterministic

##### Radioactive patient release

##### Workplace safety, ergonomics, electrical, and infection control

#### 3.5.4 Patient dosimetry

##### Internal radionuclide dosimetry

1. MIRD formalism
2. Organ and effective dose
3. Therapy treatment planning
4. Fetal dose
5. Considerations for pregnant and nursing patients
6. Considerations for pediatric patients

##### CT dosimetry

1. Dose length product
2. Fetal and organ dose

#### 3.5.5 Data science and informatics

##### Receiver operator characteristic performance

##### 3.5.5.1 Image registration and similarity metrics

##### Image processing

1. Fusion display
2. Maximum/Minimum/Average intensity projection
3. Contrast enhancement

##### Imaging statistics

1. Signal‐to‐noise ratio
2. Contrast‐to‐noise ratio
3. Statistical distributions

##### Applications of machine learning and artificial intelligence

##### Computer‐aided detection and diagnosis

##### Display performance and viewing conditions

1. Measures of luminance and illuminance
2. Just Noticeable Difference index and Barten model
3. DICOM grayscale standard display function
4. Acquisition and workstation display periodic quality control and annual assessments

##### Radiology Information system

##### Enterprise storage and display systems

1. Picture archiving and communications systems
2. Network attached storage servers, distributed licensing, and client display

##### DICOM communications and format standards

#### 3.5.6 Radionuclide and radiopharmaceutical production

##### Hot lab design and operation

##### Radionuclide characteristics

##### Radionuclide production

1. Reactors
2. Generators
3. Cyclotrons

##### Electives

1. Cyclotron design and operation
2. Radiopharmaceutical compounding and quality control

#### 3.5.7 Clinical studies

##### Clinical use of quantitative metrics

1. Ejection fraction
2. Glomerular filtration rate
3. Percent uptake
4. Kinetic analysis

##### Diagnostic procedures

1. Brain
2. Cardiac
3. Renal
4. Gastrointestinal
5. Skeletal
6. Pulmonary
7. Oncology
8. Endocrinology

##### Therapeutic procedures

1. Radiopharmaceutical
2. Radionuclide
3. Radioembolization

## 4 GUIDELINES FOR RADIATION ONCOLOGY PHYSICS RESIDENCY TRAINING PROGRAMS

### 4.1 Training content

Training programs in radiation oncology physics should demonstrate a culture of safety while providing instructional opportunities for commissioning, calibration, output and monitoring of external photon irradiation, electron therapy, and particle therapy when applicable, brachytherapy procedures, treatment planning techniques, regulatory compliance, quality assurance, and radiation safety. Areas of competence should reference accepted clinical practices and techniques which may include but are not limited to published reports by international, national, and AAPM committees or work groups, peer‐reviewed literature, textbooks, and consensus documents from leading professional organizations.

### 4.2 Facilities

Adequate space and appropriate resources must be available for the conduct of a good medical physics practice and clinical training program. At a minimum, the following resources are required to ensure that adequate therapy modalities and supporting systems are available for observation and training:

1. Megavoltage machine that includes
  - photon beams
  - electron beams
  - multileaf collimator
  - integrated imaging capability;
2. CT scanner used for radiation therapy simulation;
3. Equipment required to perform interstitial and intracavitary brachytherapy procedures, including a high dose rate afterloading treatment unit;
4. Computerized 3D external beam treatment planning system (TPS) employing modern model‐based and/or Monte Carlo calculation algorithms; and
5. Physics dosimetry laboratory housing various dosimeters and phantoms for measurement and calibration including ionization chambers, in vivo dosimeters, anthropomorphic phantoms, solid water, and water scanning systems for measurement and calibration.

### 4.3 Clinical resources

The training program in radiation oncology physics must provide access to a sufficient volume and variety of cancer patients for an adequate resident experience. The number of new external beam, brachytherapy, and special‐procedure patients treated per year should be sufficient to permit a resident to become competent and proficient in radiation oncology physics. It would be satisfactory to gain these experiences through arrangements made with other institutions for the resident to receive training on specialized equipment or treatment procedures that are not performed at the host training facility.

### 4.4 Training requirements

Upon completing a radiation oncology physics residency program, the resident should demonstrate proficiency and competency in the following areas:

1. Assessments of therapeutic systems
2. Understanding and technical knowledge of therapeutic systems
3. Safety culture
4. Professional interaction and leadership
5. Components of clinical practice
6. Professional consultation
7. Clinical procedure observations
8. Risk management
9. Regulatory compliance
10. Practice accreditation.

The program should develop a written list of objectives for or expectations of residents that should be presented to and discussed with residents. A list of specific areas of competence recommended for clinical medical physics in radiation oncology physics is provided below. Sample templates for presenting these objectives are provided in Appendix B; however, at this time, there are no standardized criteria or scales for evaluating residents’ performance during their clinical rotations. Participation in clinical research in radiation oncology physics is recommended.

Under Section 4.5, areas of competence marked as electives are topics that may not be available to all training programs. Clinical areas of competence developed in these topic areas are considered optional. However, there may be a need to develop areas of competence based on medical knowledge required to meet a level of understanding sufficient for the resident to complete their chosen subspecialty certification.

### 4.5 Expected areas of competence for clinical medical physicists in radiation oncology

#### 4.5.1 Detectors and dosimeters

##### General

1. Radiation detector applications
  - Beam measurement
  - Calibration
  - Radiation survey
  - Personnel monitoring
  - End‐to‐end phantom dosimetry and external audit dosimetry
  - In vivo dosimetry
2. Radiation detectors properties
  - Theory and mechanism of operation
  - Geometric, energy, and environmental dependencies
  - Dimensions
  - Useful clinical range
  - Ancillary supporting equipment
3. Commissioning and QA of detectors and dosimeters

##### Gas ionization chambers filled detectors ionization chamber arrays

1. Gas ionization chambers
2. Ionization chamber arrays
3. Dose calibrators and well chambers
4. Proportional counters
5. Geiger–Muller detectors

##### Solid state detectors

1. Thermoluminescent Dosimeters
2. Optically Stimulated Luminescent Dosimeters
3. Diamond Detectors
4. Metal Oxide Semiconductor Field Effect Transistor Detectors

##### Diodes

1. In vivo dosimetry
2. Small field dosimetry

##### Film

1. Radiochromic film
2. Film calibration

##### Scintillators

##### Liquid filled ionization chamber arrays

##### Other detectors

#### 4.5.2 Imaging for radiation oncology physics

##### Planar imaging and exposure measurements

1. Planar radiography
2. Fluoroscopy
3. Megavoltage portal imaging

##### CT imaging and exposure measurements

1. Helical CT
2. Four‐dimensional CT
3. Cone beam CT

##### Surface imaging

##### PET

##### SPECT

##### MRI

##### US

#### 4.5.3 Data science and informatics

##### Receiver operator characteristic performance

##### Image registration and similarity metrics

##### Image processing

1. Fusion display
2. Maximum/Minimum/Average intensity projection
3. Contrast enhancement
4. Quantitative imaging and signal mapping

##### Imaging statistics

1. Signal‐to‐noise ratio
2. Contrast‐to‐noise ratio
3. Statistical distributions

##### Computer‐aided detection and diagnosis

##### Applications of artificial intelligence and machine learning

##### Enterprise storage and display systems

1. Picture archiving and communications systems (PACS)
2. Network attached storage servers, distributed licensing, and client display

##### DICOM communications and formatting standards

##### Record and verify system

##### Clinical trial design and statistical methodology

##### Electives

1. Uniform analysis standards
2. Relational and graphical databases

#### 4.5.4 External beam radiation therapy

##### Acceptance

1. Tolerances and specifications for assessments
2. Dosimetric beam evaluation
3. Mechanical evaluation
4. Imaging systems evaluation
5. Ancillary systems evaluation
6. Safety systems

##### Commissioning

1. Beam data requirements
2. Phantoms
3. Detectors
4. Data collection methods
5. Data processing
6. Absolute output
7. End‐to‐end testing
8. Setting baselines

##### Evaluation of treatment planning systems

1. Modeling data in treatment planning systems
2. Modeling ancillary equipment
3. Validation
4. Baselines

##### Simulation for electron beam radiation therapy

1. Patient positioning and immobilization
2. Motion management
3. Imaging modalities and equipment for simulation
4. Virtual simulation
5. Requirements for treatment

##### Treatment planning and dose calculations

1. Manual monitor unit calculations
2. Imaging modalities and fusions for planning
3. Definition of target volume and critical structures
4. Normal tissue and critical structure contouring
5. Target volume definitions
6. Normal tissue tolerances and radiation side effects
7. Photon and electron beam properties
8. Considerations for implanted devices
9. Planning considerations for various anatomical sites
10. Treatment planning techniques
  - Electron planning
  - Two‐dimensional photon planning
  - 3D conformal radiation therapy planning
  - Intensity modulated radiation therapy and volumetric modulated arc therapy planning
  - Stereotactic radiosurgery planning and Stereotactic ablative radiation therapy
  - Special procedure planning including total skin electron therapy and total body irradiation
  - Adaptive planning
11. Dose calculation
  - Correction‐based algorithms
  - Correction for irregular fields
  - Correction for irregular surfaces
  - Correction for heterogeneity
  - Model‐based algorithms
  - Monte Carlo algorithms

##### Treatment equipment and techniques

1. General concepts
  - Linear accelerator (linac) design
  - Isocentric and nonisocentric configurations
2. 3D conformal radiation therapy
  - Fundamentals of 3D planning
  - Beam modifiers
  - Multileaf collimator design
  - Sources‐axis distance and source‐surface distance setup
  - Field matching
3. Electrons
  - Beam collimation including patient specific shielding
  - Field matching
  - Virtual source distance and effective source‐to‐skin surface
  - x‐Ray contamination
4. Intensity modulated radiation therapy
  - Fixed gantry delivery
  - Rotational delivery
  - Inverse optimization algorithms
  - Multileaf collimator dosimetric considerations
5. Stereotactic radiosurgery
  - Rationale for treatments
  - Radiobiological considerations
  - Linac and Co‐60 configurations
  - Small field dosimetry
  - Quality assurance requirements
  - Additional quality assurance requirements
  - Stereotactic cone
  - Frame‐based versus frameless treatments
  - Target imaging and localization
  - Motion management
6. Stereotactic body radiation therapy (SBRT)
  - Rationale for treatments
  - Radiobiological considerations
  - Small field dosimetry
  - Quality assurance requirements
  - Target imaging and localization
  - Motion management
7. Total body irradiation
  - Rationale for treatments
  - Prescriptions and delivery techniques
  - Commissioning considerations
  - Beam modifiers
  - Patient simulation
  - In vivo dosimetry
8. Total skin electron therapy (TSET)
  - Rationale for treatments
  - Delivery techniques
  - TSET Beam characteristics
  - Commissioning considerations
  - Boost field determination
  - In vivo dosimetry and treatment delivery quality checks
9. Adaptive therapy
  - Rational for treatments
  - Acceptance and commission considerations
  - Delivery systems
  - Offline versus online
  - Machine specific quality assurance considerations
10. Proton therapy
  - Proton interaction in tissue
  - Rationale for treatments
  - Delivery systems
  - Acceptance and commissioning considerations
  - Major components of beam delivery system: scattering and scanning
  - Range modulators, beam compensators, and apertures
  - Dosimetry calibration protocols
  - Uncertainties in range, density, motion and associated dosimetric effects
  - Proton‐specific treatment planning considerations
  - Proton‐specific quality assurance devices and activities
11. Electives
  - Heavy ion therapy
  - Neutron therapy
  - MR linac
  - Biologically guided radiation therapy
  - kV and orthovoltage x‐ray therapy
  - Nonisocentric SRS/SBRT systems
  - Electron intraoperative therapy
  - Ring‐gantry MV linac

##### Image guidance and motion management for treatment

1. In‐room x‐ray imaging
2. US
3. Surface guidance
4. Electromagnetic beacons
5. MRI
6. Imaging dose and safety considerations

##### Treatment quality assurance

1. Measurement‐based patient‐specific quality assurance systems
2. Film and chamber
  - Ionization chamber array
  - Diode array
  - Electronic portal imaging device
3. Calculation‐based patient‐specific quality assurance
  - Log‐file
  - Monte Carlo
4. Patient‐specific quality assurance analysis
  - Gamma analysis
  - Recommended tolerances and passing rates
5. Physics plan and chart reviews
  - Initial plan review
  - Secondary monitor unit calculation
  - Weekly chart check
  - End‐of‐treatment chart check
6. Device and routine equipment quality assurance procedures
  - Dosimetry and delivery system
  - Mechanical system
  - Imaging guidance system
  - Simulation system
  - Safety systems
  - Supporting equipment and their calibration
  - Treatment planning system
  - Recommended testing frequencies and tolerances

#### 4.5.5 Brachytherapy

##### Calibration, safety, hazards, and regulations

1. Well detector theory, operation, and calibration
2. Source strength, decay characteristics, construction, and geometry
3. Source calibration and verification
4. Personal protective equipment and handling
5. Emergency procedures training
6. Patient release
7. Regulatory bodies

##### Acceptance testing and commissioning

1. Establishing program and procedures
2. Source(s)
3. Treatment planning system
4. Applicators
5. Remote afterloader

##### Treatment techniques

1. Intracavitary
2. Interstitial
3. Surface

##### Simulation

1. Imaging modality selection, planning, and guidance
2. Immobilization techniques for applicators
3. Applicator position verification and channel delineation

##### Treatment planning and dose calculation

1. Critical organs and target delineation
2. Normal tissue tolerances and radiation side effects
3. Applicator reconstruction
4. Dose calculation formalism with or without heterogeneity correction
5. Geometric and volumetric dose optimization
6. Independent verification of treatment plan
7. Postimplant dosimetry
8. Decay calculations
9. Radiobiological considerations

##### Unsealed source therapy

1. Radionuclide
2. Radioembolization
3. Radiopharmaceutical

##### Electives

1. Intraoperative brachytherapy
2. Electronic brachytherapy
3. Plaque brachytherapy
4. Intraluminal brachytherapy
5. Intravascular brachytherapy

##### Quality assurance

1. Quality management and process quality control
2. Patient‐specific quality assurance
3. Routine equipment quality assurance

#### 4.5.6 General safety and radiation protection

##### Regulatory agencies, jurisdiction, and publications

##### Advisory bodies

##### Storage of Radioactive isotopes and materials

##### Radioactive material transport, receiving, and disposal

##### Protection standards and practices

1. Dose quantities and units
2. Regulatory limits and usage regulations

##### Protection practices and contamination control

1. Radiation safety program
2. Quality management
3. Historical accidents
4. Guidelines and safety principles

##### Medical use regulations

1. Authorized user
2. Written directive
3. Medical event
4. Patient release

##### Shielding for external beam, brachytherapy, and simulators

1. Shielding design goals
2. Facility design
3. Barrier thickness calculations
4. Radiation safety survey
5. Considerations for hybrid systems

##### Radiation risks

1. Stochastic
2. Deterministic
3. Models of risk assessment
4. Fetal dose
5. Considerations for pregnant and nursing patients

##### Personnel monitoring

1. Regulatory limits and badging
2. Personal dosimeters

#### 4.5.7 Radiobiology

##### Radiation cell killing models

##### Dose and fractionation for normal and tumor tissues

##### Tumor control probability

##### Normal tissue complication probability

##### Relative biological effectiveness

##### R's—Repair, repopulation, redistribution, reoxygenation

##### α/β ratio, linear‐quadratic calculations, equivalent dose

##### Clinical applications of radiobiology

#### 4.5.8 Quality management

##### Quality management program structure and personnel

##### Practice accreditation

##### Safety strategies

##### Retrospective risk analysis methods (e.g., incident learning)

##### Prospective risk analysis methods (e.g., failure mode and effects analysis review)

##### Safety checklists

##### Safety culture

##### Quality audit

## AUTHOR CONTRIBUTIONS

**Joseph P. Dugas, William D. Erwin, David P. Gierga, Darryl G. Kaurin, Stephanie M. Leon, Ho‐Ling Anthony Liu, Zheng Feng Lu, Alphonso W. Magri, Jonathon A. Nye, Lynn N. Rill, Leah K. Schubert, Beth A. Schueler, Amy Shu‐Jung Yu, Irina Vergalasova, John Vetter, Brian Wichman, Dandan Zheng**: Conceptualization (equal), data curation writing—original draft preparation (equal), and writing—review and editing (equal). **Jonathon A. Nye**: Writing—review and editing (lead).

## CONFLICT OF INTEREST STATEMENT

The members of the WGMPRT listed below attest that they have no potential conflicts of interest related to the subject matter or materials presented in this document. The Chair of the Work Group on Periodic Review of Medical Physics Residency Training (WGMPRT) has reviewed the conflicts of interest statement on file for each member of the WGMPRT and determined that the disclosure of potential conflicts of interest is an adequate management plan. Disclosures of potential conflicts of interest for each member of the WGMPRT are found at the close of this document.

## APPENDIX A RESIDENT, STAFF, AND PROGRAM EVALUATION FORMS

### A.1

Please note: The forms shown below are intended to be samples to assist program directors in continually improving their residency training programs. The forms have emerged from multiple residency training programs and should be modified to fit the needs of individual programs. There are currently no standardized criteria or scales for evaluating staff and residents.

The evaluation forms include the following:

1. Resident evaluation form
2. Staff evaluation form
3. Program evaluation form

## APPENDIX B CLINICAL OBJECTIVES FORMS FOR IMAGING, NUCLEAR MEDICINE, AND RADIATION ONCOLOGY

### B.1

Please note: The forms below are intended to be samples to assist program directors in the continual improvement of their residency training programs. The forms have emerged from multiple residency training programs and should be modified to fit the needs of individual programs. There are currently no standardized criteria or scales for evaluating resident performance during clinical rotations.

The clinical objective forms include the following:

1. Medical physics residents in imaging evaluation
2. Medical physics residents in nuclear medicine evaluation
3. Medical physics residents in radiation oncology evaluation

## APPENDIX C COMBINING IMAGING AND NUCLEAR MEDICINE RESIDENCIES

### C1 Introduction

This appendix describes those areas of competence common to imaging and nuclear medicine residency training programs, areas of competence in the imaging residency to be augmented for nuclear medicine physics, and areas of competence unique to nuclear medicine physics that must be completed to provide a combined imaging and nuclear medicine physics residency. At the completion of the combined residency training program, the resident shall demonstrate competency in all subjects in Sections 2 and 3.

### C2 Common areas of competence

General areas of competence associated with the program environment (Section 1) and overall adjustment to the residency environment are common to both imaging and nuclear medicine physics residency training programs. The total time devoted to mastering the areas of competence in Sections C3–C5 would be equivalent to a minimum of a 24‐month nuclear medicine residency.

### C3 Areas of competence completely covered in imaging physics

The following areas of competence are covered under the imaging physics residency training program and fulfill the requirements for a nuclear medicine physics program, either completely or with minor augmentation:

1. CT (Section 2.5.3)
2. MRI (Section 2.5.6)
3. Data science and informatics (Section 2.5.8).

### C4 Areas of competence requiring augmentation for nuclear medicine physics

The following imaging areas of competence shall be augmented to address specific nuclear medicine physics requirements:

1. Nuclear medicine and PET (Section 2.5.7)
2. General safety and radiation protection (Section 2.5.9).

### C5 Areas of competence unique to nuclear medicine

The following areas of competence unique to nuclear medicine physics shall be addressed and completed:

1. Planar imaging (Section 3.5.1)
2. SPECT and SPECT/CT (Section 3.5.1)
3. PET/CT (Section 3.5.1)
4. Nonimaging devices (Section 3.5.2)
5. Patient dosimetry (Section 3.5)
6. Radionuclide and radiopharmaceutical production (Section 3.5.6)
7. Clinical studies (Section 3.5.7).

### C6 Conclusion

Imaging physics residency training program staff are encouraged to explore options for providing a combined imaging and nuclear medicine physics residency. This may be accomplished in an additional year (following completion of an imaging residency) using staff from the residency training program who meet the personnel requirements described in Section 1.2.9. Alternatively, the additional year of nuclear medicine training may be provided at another facility with adjunct staff and facilities that meet the residency training program requirements described in Section 1.2. This arrangement would be governed by an affiliation agreement that specifies that residency training program staff are responsible for ensuring that all program requirements are met. In either case, the program staff shall include at least one nuclear medicine physicist certified by an appropriate certifying board and a nuclear medicine physician certified by the American Board of Radiology or its equivalent, depending on the subfield of medical physics in which the program specializes.

## References

[1] American Association of Physicists in Medicine . Public & media. Accessed July 28, 2022. https://w3.aapm.org/media

[2] Prisciandaro JI , Willis CE , Burmeister JW , et al. Essentials and guidelines for clinical medical physics residency training programs. AAPM; 2013. Report No. 249. Accessed May 8, 2023. https://www.aapm.org/pubs/reports

[3] Lane RG , Stevens DM , Gibbons JP , et al. Essentials and guidelines for hospital‐based medical physics residency training programs. AAPM; 2006. Report No. 90. Accessed June 10, 2023. https://www.aapm.org/pubs/reports

[4] Sternick ES , Evans RG , Morin RL , et al. Essentials and guidelines for hospital based medical physics residency training programs. AAPM; 1990. Report No. 036. Accessed February 12, 2023. https://www.aapm.org/pubs/reports

[5] Herman MG , Harms TA , Hogstrom KR , et al. Alternative clinical medical physics training pathways for medical physicists. Report of AAPM Task Group 133. 2008. Accessed June 20, 2022. https://www.aapm.org/pubs/reports

[6] American Board of Radiology . ABR residency leave policy. Accessed May 18, 2023. https://www.theabr.org/exam‐details/residency‐leave‐policy

[7] AAPM . Academic program recommendations for graduate degrees in medical physics. AAPM Report No. 365. Accessed October 2, 2023. https://www.aapm.org/pubs/reports

[8] Serago CF , Burmeister JW , Dunscombe PB , et al. Recommended ethics curriculum for medical physics graduate and residency programs: report of Task Group 159. Med Phys. 2010;37:4495‐4500. 20879608 10.1118/1.3451116

[9] American Association of Physicists in Medicine . AAPM code of ethics, PP 24‐A. Accessed July 28, 2022. https://www.aapm.org/policies

[10] ABR/ACR/RSNA/AAPM/ASTRO/ARR/ARS, online modules on ethics and professionalism. Accessed July 28, 2022. http://www.aapm.org/education/onlinemodules.asp

[11] American Association for Physicists in Medicine . Medical physics leadership academy. Accessed July 28, 2022. https://w3.aapm.org/leadership/index.php?od1n

[12] American Association of Physicists in Medicine . Diversity Statement, AP 132‐A. Accessed July 28, 2022. https://www.aapm.org/policies

[13] American Association of Physicists in Medicine . Medical Physics 3.0. Accessed July 28, 2022. https://mp30.aapm.org 10.1002/j.2473-4209.2012.tb00183.x 28525103

[14] American Board of Radiology . ABMS and ACGME: six core competencies for initial certification. Beam. 2022 51(4):8.

[15] Pfeiffer D , Al‐Hallaq H , Halvorsen P , et al. AAPM medical physics practice guideline 3.b.: levels of supervision for medical physicists in clinical training. J Appl Clin Med Phys. 2021;22:11‐15. 10.1002/acm2.13258 PMC8200506 34018313

[16] Cate Olleten , Chen HuijuCarrie , Hoff ReinierG , Peters Harm , Bok Harold , Schaaf Mariekevander . Curriculum development for the workplace using entrustable professional activities (EPAs): AMEE Guide No. 99. Med Teach. 2015;37:983‐1002. 26172347 10.3109/0142159X.2015.1060308

[17] Edgar L , McLean S , Hogan SO , Hamstra S , Holmboe ES . The Milestones Guidebook, Version 2020. (2023, May 17) 2022. https://www.acgme.org/what‐we‐do/accreditation/milestones/resources/

[18] Holmboe ES , Iobst WF . (2023, May 17) Assessment Guidebook. Accessed Jan 20 https://www.acgme.org/what‐we‐do/accreditation/milestones/resources/, 2022.

